# The apoptosome molecular timer synergises with XIAP to suppress apoptosis execution and contributes to prognosticating survival in colorectal cancer

**DOI:** 10.1038/s41418-020-0545-9

**Published:** 2020-04-27

**Authors:** Gavin Fullstone, Tabea L. Bauer, Cristiano Guttà, Manuela Salvucci, Jochen H. M. Prehn, Markus Rehm

**Affiliations:** 1 Institute for Cell Biology and Immunology, Allmandring 31, 70569 Stuttgart, Germany; 2 Stuttgart Research Centre Systems Biology, Nobelstraße 15, 70569 Stuttgart, Germany; 3 SimTech Cluster of Excellence, Pfaffenwaldring 5a, 70569 Stuttgart, Germany; 4 Department of Physiology and Medical Physics, Royal College of Surgeons in Ireland, Dublin 2, Ireland; 5 Centre for Systems Medicine, Royal College of Surgeons in Ireland, Dublin 2, Ireland

**Keywords:** Cancer, Cell biology

## Abstract

The execution phase of apoptosis is a critical process in programmed cell death in response to a multitude of cellular stresses. A crucial component of this pathway is the apoptosome, a platform for the activation of pro-caspase 9 (PC9). Recent findings have shown that autocleavage of PC9 to Caspase 9 (C9) p35/p12 not only permits XIAP-mediated C9 inhibition but also temporally shuts down apoptosome activity, forming a molecular timer. In order to delineate the combined contributions of XIAP and the apoptosome molecular timer to apoptosis execution we utilised a systems modelling approach. We demonstrate that cooperative recruitment of PC9 to the apoptosome, based on existing PC9-apoptosome interaction data, is important for efficient formation of PC9 homodimers, autocatalytic cleavage and dual regulation by XIAP and the molecular timer across biologically relevant PC9 and APAF1 concentrations. Screening physiologically relevant concentration ranges of apoptotic proteins, we discovered that the molecular timer can prevent apoptosis execution in specific scenarios after complete or partial mitochondrial outer membrane permeabilisation (MOMP). Furthermore, its ability to prevent apoptosis is intricately tied to a synergistic combination with XIAP. Finally, we demonstrate that simulations of these processes are prognostic of survival in stage III colorectal cancer and that the molecular timer may promote apoptosis resistance in a subset of patients. Based on our findings, we postulate that the physiological function of the molecular timer is to aid XIAP in the shutdown of caspase-mediated apoptosis execution. This shutdown potentially facilitates switching to pro-inflammatory caspase-independent responses subsequent to Bax/Bak pore formation.

## Introduction

Apoptosis is the process of controlled cell death, integral for development and maintaining homoeostasis. Its dysregulation has been implicated in cancer, autoimmune and neurodegenerative diseases [1]. Apoptosis signalling is strictly controlled by a family of cysteine aspartate specific proteases (Caspases), which are expressed as inactive zymogens (Pro-Caspases) [2]. The execution phase of apoptosis is triggered in response to multiple extrinsic and intrinsic stress signals by Bax/Bak-mediated mitochondrial outer membrane permeabilisation (MOMP) [3]. MOMP allows the release of the mitochondrial proteins cytochrome c (CytC) and second mitochondrial-derived activator of caspases (SMAC) into the cytosol, initiating the apoptosis execution phase cascade [3]. CytC interacts with apoptotic peptidase activating factor 1 (APAF1) and along with ATP causes its oligomerisation into a heptameric complex called the apoptosome. Pro-Caspase 9 (PC9) is recruited to the apoptosome platform, which is strictly necessary for PC9’s activity in cleavage of the apoptosis executioners pro-caspase 3/7 (PC3/7) and for PC9 autocatalytic cleavage to the caspase 9 (C9) 35/12 isoform [4]. The executioner caspase 3 (C3) mediates the cleavage of many downstream substrates, subsequently giving rise to the apoptosis phenotype including blebbing, cell shrinkage, DNA fragmentation and non-inflammatory cell death. The X-linked inhibitor of apoptosis protein (XIAP) inhibits the activity of C3 and C9-35/12 [5–7], but is in turn inhibited by SMAC released by MOMP [8, 9].

Historically, two theories were put forward for the mechanism of activation of PC9 on the apoptosome. The first is that the apoptosome facilitates PC9 homodimerisation by increasing the local concentration of the zymogen (the induced-proximity model) [10, 11] and the second is it induces a conformational change in the monomeric enzyme leading to activation (allosteric activation model) [12–14]. We previously have applied a systems approach which supported allosteric activation owing to insufficient C3-substrate cleavage to reproduce experimental data, obtained using Förster resonance energy transfer (FRET)-based reporters, when PC9 is activated exclusively as a homodimer [15]. In a recent study, it was concluded that PC9 is active in cleaving PC3 as a homodimer, but can also be activated by forming a heterodimer with the nucleotide-binding oligomerisation domain of APAF1 [16]. However, autocatalytic cleavage of PC9 to C9-35/12 occurs exclusively in a PC9 homodimer [16]. This cleavage is not necessary for its activity, but appears to play an integral role in regulation of the apoptosome activity. Previously, it has been demonstrated that autocatalytic cleavage at D^315^ exposes a short peptide sequence required for the efficient inhibition of C9-35/12 by XIAP [9, 17, 18]. Moreover, it has been proposed that the autocleavage of PC9 to C9-35/12 initiates a molecular timer, where the activity of the apoptosome temporally switches off, due to the poor affinity of C9-35/12 to the apoptosome [18]. In the work of Malladi and colleagues, it was proposed that this may protect cells that undergo aberrant partial release of pro-apoptotic factors from the mitochondria. Interestingly, such a process has been recently described where specific doses of the Bcl-2 inhibitor ABT-737 or infections caused only a minority of mitochondria to undergo MOMP, termed minority MOMP (minMOMP) [19, 20]. Rather than culminating in cell death, minMOMP caused sublethal C3-activation leading to pro-tumourigenic and inflammatory responses. Despite this finding, the molecular timer effect has only been shown in reconstituted apoptosome experiments using recombinant proteins or in knockout cells reconstituted with non-cleavable mutants of PC9 [16, 18]. Therefore, it is as-of-yet unknown whether the molecular timer is able to actively prevent apoptosis execution under physiological conditions. Furthermore, delineating the contributions of the molecular timer from XIAP-inhibition is experimentally inaccessible as both processes are triggered by the autocatalytic cleavage of PC9 [9, 17, 18].

In order to elucidate the physiological contribution of the molecular timer in apoptosis, we set out to implement new findings in apoptosome recruitment, activation and processing of PC9 in a systems model of apoptosis execution. We demonstrate that a cooperative model of PC9 recruitment to the apoptosome is necessary for robustly reproducing experimental data on PC9 cleavage rates, the molecular timer and XIAP-inhibition of the apoptosome. We then utilise this new model, along with a variant where the molecular timer is effectively turned off, to broadly screen the complete range of typical protein expression levels to determine circumstances when the molecular timer can actively prevent apoptosis execution. Finally, we demonstrate that our model is prognostic for survival of colorectal cancer patients and that the molecular timer contributes to apoptosis resistance in these patients.

## Materials and methods

### Model implementation and parameterisation

All models were implemented as systems of ordinary differential equations (ODE) based on our previously published ApoptoAll model, with minor alterations [15]. An overview of all reactions and parameterisation of the models is described in Supplementary Text 1. Minimal MOMP conditions were modelled as release of 5% of the total mitochondrial SMAC and CytC. The ODE system was implemented and solved using MATLAB and Statistics Toolbox Release 2016b, (The MathWorks, Inc., Natick, MA, USA) and the solver module ode15s with an absolute tolerance of 10^−19^ and a relative tolerance of 10^−8^.

### Extraction of k_on_ and k_off_ values from SPR traces

The dissociation curves of different PC9 variants were extracted from the surface plasmon resonance (SPR) curves published by Wu et al. using Engauge Digitizer v10.11 (M. Mitchell, B. Muftakhidinov, T. Winchen, B. van Schaik, A. Wilms, Z. Jędrzejewski-Szmek et al., 2019, 10.5281/zenodo.1472917) [16]. Subsequently extracted points were subjected to one phase decay non-linear regression using GraphPad Prism 6 (GraphPad Software Inc., San Diego, CA, USA). The obtained dissociation halftimes from the curve fitting of different PC9 variants were used to determine kinetics values of PC9 and C9 binding to the apoptosome. *k*_off_ values were calculated directly from the determined halftimes (*t*_1/2_) using the relation:

1 $${\it{k}}_{{\it{\mathrm{off}}}} = \frac{{{\mathrm{ln}}\left( 2 \right)}}{{{\it{t}}_{1/2}}}.$$

The *K*_D_ values published by Wu et al. and the calculated *k*_off_ values were then used to obtain the *k*_on_ values using the relation [16]:

2 $$k_{{\mathrm{on}}} = \frac{{k_{{\mathrm{off}}}}}{{K_{\mathrm{D}}}}.$$

### Parameter estimation

Cooperative recruitment was implemented by performing a global parameter estimation using least squares regression to determine kinetic values for primary binding of PC9, cooperative secondary binding of PC9 and binding of C9 to the apoptosome. Full details and justification are included in Supplementary Text 2. In brief, SPR data were used to set the bounds of primary and secondary binding of PC9 to the apoptosome with primary binding assumed weaker than the SPR data (*k*_on(primary)_ < *k*_on(SPR)_, *k*_off(primary)_ > *k*_off(SPR)_) and secondary binding assumed stronger (*k*_on(secondary)_ > *k*_on(SPR)_, *k*_off(secondary)_ < *k*_off(SPR)_) to reflect the likely mixture of primary and secondary binding in SPR readouts. Experimental C3-substrate cleavage and molecular timer data were used as training data with equal weighting [18, 21]. C3-substrate cleavage was based on cleavage of FRET probes in HeLa cells fitted to a Boltzmann curve, as described previously. Experimental molecular timer data were extracted from Wu et al. using ImageJ (Wayne Rasband, National Institute of Health, USA) [16].

### Survival curves

For survival analysis, 1000 different combinations of APAF1, PC9, PC3, XIAP and SMAC as initial concentrations were generated by randomly sampling from physiologically relevant protein expression ranges. Each condition was simulated using four different conditions, normal, no XIAP, no molecular timer and no molecular timer & no XIAP. Death events were defined by inclusion of an event function into the ODE solver to determine the time point where C3-substrate cleavage >25%. If no death event was recorded within 4 h of simulation time, i.e. C3-substrate cleavage ≤25% for the duration of the simulation, then a censored event was recorded at 240 min. Webb’s fractional product was calculated from the fraction of surviving cells in the absence of XIAP, the molecular timer or both at 15, 30, 60 and 240 min, normalised against the standard model with XIAP and the molecular timer present, using the formula:

3 $$	{{{\rm{Synergy}}\,{\rm{Score}}}} =\\ 	\!\frac{{\left({{{{\rm{Survival}}_{{\rm{No}}\,{\rm{XIAP}}\,\& \,{\rm{No}}\,{\rm{Mol}}\,{\rm{Timer}}}}} \div {{{\rm{Survival}}_{{\rm{Normal}}}}}} \right)}}{{\left({{{\rm{Survival}}_{{\rm{No}}\,{\rm{XIAP}}}} \div {{{\rm{Survival}}_{{\rm{Normal}}}}}} \right) \times \left( {{{\rm{Survival}}_{{\rm{No}}\,{\rm{Mol}}\,{\rm{Timer}}}} \div {{{\rm{Survival}}_{{\rm{Normal}}}}}} \right)}}.$$

Scores of <0.9 were considered synergistic.

### IETDase activity calculation

IETDase activity at time *t* was calculated from the concentration of active PC9-apoptosome ([Active PC9]) and active C9-apoptosome ([Active C9]) complexes using the kinetic values for their respective cleavage of PC3 (k_PC9_ and k_C9_):

4 $${\mathrm{IETDase}}_{{\mathrm{Total}}}\left( {{t}} \right) = {\mathrm{k}}_{{\mathrm{PC}}9}\left[ {{\mathrm{Active}}\,{\mathrm{PC}}9\left( {\mathrm{t}} \right)} \right] + {\mathrm{k}}_{{\mathrm{C}}9}\left[ {{\mathrm{Active}}\,{\mathrm{C}}9\left( {\mathrm{t}} \right)} \right].$$

### Screening for molecular timer activity

Screening of the concentration-dependent activity of the molecular timer was performed by screening the entire range of physiologically relevant PC9 and APAF1 concentrations as a linearly distributed 25 × 25 concentration matrix. Simulations were performed for 4 h before determination of the max activity, time to max activity, integrated activity and half-life of activity decay. Integrated activity was calculated by using the trapz function in Matlab for approximation of the area under the curve (AUC) for the IETDase activity. The half-time of decay was calculated using the Matlab nlinfit function for non-linear fitting to fit the IETDase activity to the one phase decay equation:

5 $${\it{y}} = \left( {{\it{y}}_0 - {\mathrm{Plateau}}} \right) \times {\it{e}}^{ - {\it{kx}}} + {\mathrm{Plateau}},$$

where *y*_0_ is equal to the activity at time 0, in this case the maximum IETDase activity. The half-life plotted was obtained from the fitted coefficient k by:

6 $${\it{t}}_{1/2} = \frac{{{\mathrm{ln}}\left( 2 \right)}}{{\it{k}}}.$$

Parameter screening for the effect of the molecular timer was performed by randomly sampling each protein from pre-determined physiological concentration ranges to make entirely unique combinations (*n* = 1000 for survival curves, *n* = 100,000 ROC curves). As CytC and ATP are generally considered non-limiting, we kept the values in line with concentrations previously determined in HeLa cells, 10 and 920 μM, respectively [22, 23]. Concentration ranges were run for 1 h with or without the molecular timer and C3-substrate cleavage was calculated to determine apoptosis capability. Receiver operator curves were generated in Graphpad Prism 6 using input concentrations from each class (apoptosis capable/resistant; molecular timer dependent/independent).

### Application of the model to colorectal cancer patient data

Stage III colorectal cancer patient data, including survival data and protein expression data were published previously [24]. All patients underwent 5-fluorouracil-based adjuvant chemotherapy and had clear resection margins (R0). Raw data and processing scripts are downloadable from 10.5281/zenodo.1162683 [24]. Concentrations of PC9, PC3, XIAP and SMAC were used as inputs alongside the median value of APAF1 measured previously in colorectal cancer cells of 0.123 μM [25]. Patients were split into two classes according to the predicted level of C3-substrate cleavage after 300 min of simulation, apoptosis resistant (≤25%) and apoptosis capable (>25%). Kaplan–Meier curves were plotted from disease free and overall survival data of the patients.

## Results

### Homodimerisation-mediated autocleavage of PC9 alone fails to replicate the apoptosome molecular timer and XIAP-sensitivity

**Fig. 1:**
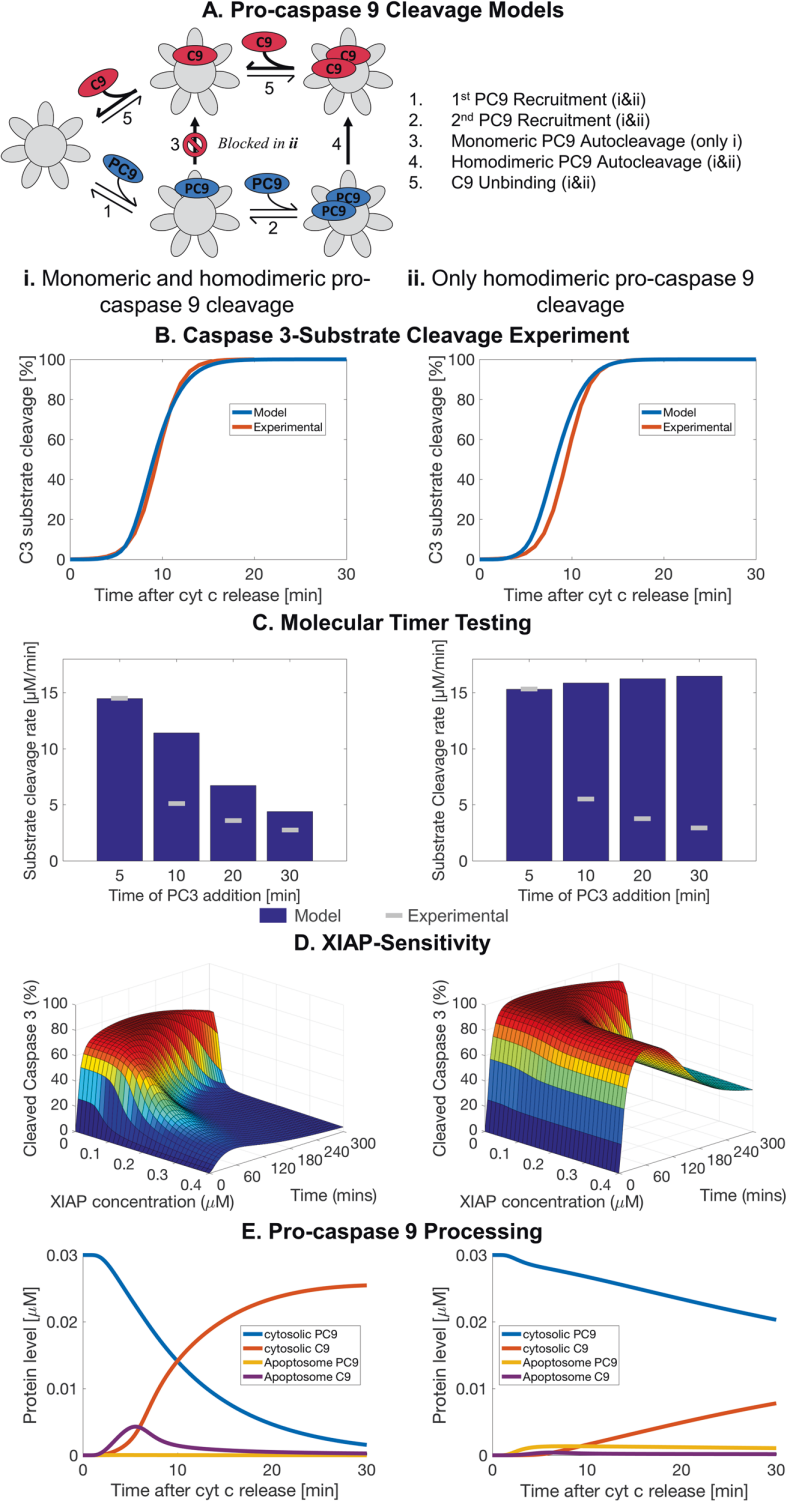
Mathematical modelling of homodimerisation-mediated autocleavage fails to replicate the apoptosome molecular timer and XIAP-sensitivity. Comparison of the apoptosis execution models with monomeric PC9 cleavage (i) and with only homodimeric PC9 cleavage (ii) (**a**). Simulation of C3-substrate cleavage in HeLa cells against experimental data derived from DEVD-FRET probes (**b**). Simulations of the molecular timer, APAF1 (0.3 μM), PC9 (0.0125 μM), ATP (1 mM) and CytC (10 μM) were incubated for 5–30 min as indicated before addition of PC3 (0.5 μM) and C3-substrate (15 μM) [18]. Values are given as the maximal rate of C3-substrate cleavage (**c**). Percentage of cleaved C3 (free and XIAP-bound) compared to total PC3+C3 after simulating HeLa cell conditions with different levels of XIAP (**d**). Percentage of PC9 and C9 over total PC9+C9 in the cytosol and at the apoptosome (**e**). Experimental data from B sourced from Rehm and colleagues [21]. Experimental data from C sourced from Malladi and colleagues [18].

Previously, we demonstrated that homodimerisation-based activation of PC9 was insufficient to explain experimental data on subsequent C3-activity and therefore we suggested that allosteric activation likely contributes to PC9 activation at the apoptosome [15]. Recently, it was demonstrated that PC9 can indeed be activated as a monomer by heterodimerisation with APAF1, but requires homodimerisation for autocatalytic cleavage to C9-35/12 [16]. We therefore postulated that removal of monomeric autocatalytic cleavage from an allosteric activation model may lead to inefficient autocatalytic cleavage (Fig. 1a). Simulating the cleavage of a C3-substrate using concentrations based on HeLa cells, the new model demonstrated similar rates of substrate cleavage to that determined experimentally using a C3-substrate FRET reporter and by our previous model (Fig. 1b). However, the model with homodimerisation-mediated cleavage only failed to replicate the temporal reduction of apoptosome activity, characteristic of the molecular timer, observed experimentally by Malladi et al. (Fig. 1c). Moreover, overexpression of XIAP failed to notably decrease the level of cleaved caspase 3 demonstrating poor inhibition of apoptosome activity (Fig. 1d). Together, this suggested that PC9 autocleavage, a necessary step for both the molecular timer and XIAP inhibition, was inadequate and the observed C3-substrate cleavage was mainly driven by PC9 monomers. This was confirmed by poor processing of PC9 (Fig. 1e and Supplementary Fig. 1ai), not matching the reported turnover rates of PC9 (Supplementary Fig. 1bi) [26–29]. Moreover, the resulting apoptosome complexes were almost exclusively populated by PC9 and the levels did not notably decrease after 5 min (Fig. 1e). Flux analysis confirmed that the rate of PC9 cleavage was relatively poor with most of the dynamic activity at the apoptosome PC9 binding and unbinding (Supplementary Fig. 1ai). Therefore, the current understanding of PC9 recruitment, dimerisation and cleavage at the apoptosome fails to replicate experimental data, the molecular timer effect and XIAP-inhibition of the apoptosome.

### Cooperative binding of PC9 to the apoptosome is essential for efficient PC9 cleavage, the apoptosome molecular timer and XIAP-sensitivity

**Fig. 2:**
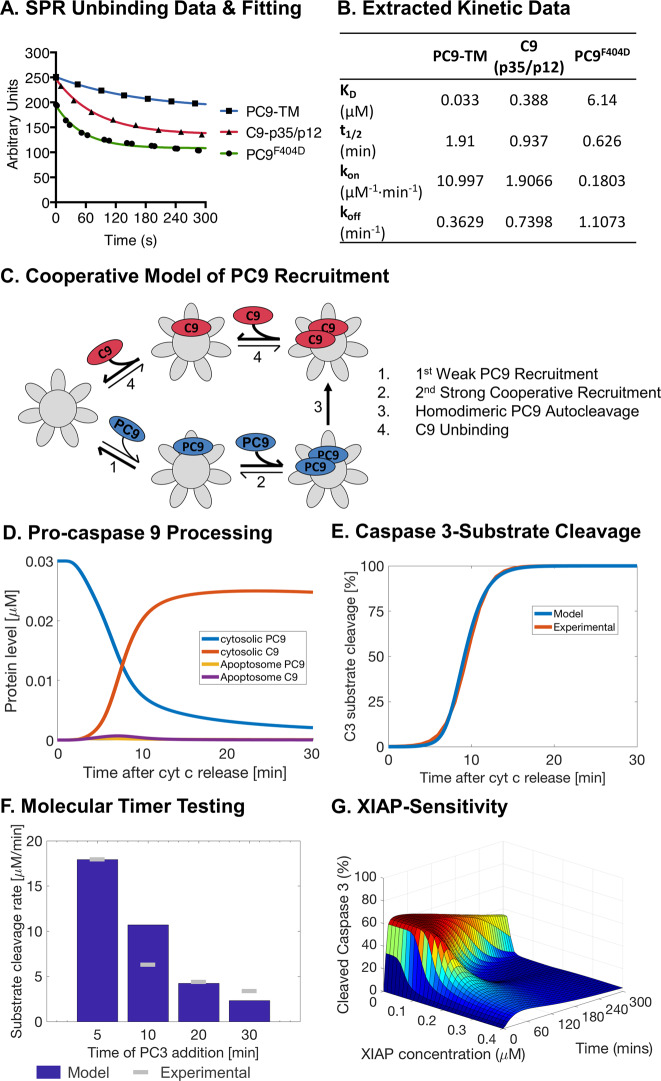
A cooperative-binding model of pro-caspase 9 recruitment to the apoptosome leads to efficient pro-caspase 9 cleavage and restoration of the molecular timer and XIAP sensitivity. SPR data points (symbols) extracted from experimental data were fitted to a one phase decay equation (lines) by a non-linear regression with all *R*^2^ > 0.98 (**a**). Binding (*k*_on_) and unbinding (*k*_off_) rates were calculated from reported dissociation coefficients (*K*_D_) and half times (*t*_1/2_) from non-linear regression in **a** (**b**). Schematic of the cooperative model of PC9 recruitment to the apoptosome, subsequent cleavage and C9 dissociation (**c**). Percentage of PC9 and C9 over total PC9+C9 in the cytosol and at the apoptosome using cooperative recruitment (**d**). Simulation of C3-substrate cleavage in HeLa cells with cooperative recruitment against experimental data derived from DEVD-FRET probes (**e**). Simulations of the molecular timer with cooperative recruitment (**f**). Percentage of cleaved caspase 3 (free and XIAP-bound) compared to total PC3+C3 after simulating HeLa cell conditions with different levels of XIAP using cooperative recruitment (**g**). Simulations and experimental data as in Fig. 1.

The classical induced-proximity model of pro-caspase activation assumes that each pro-caspase, in our case PC9, is recruited individually to an activation platform where they undergo homodimerisation due to the increase in local zymogen concentration. However, PC9 is sub-stochiometric compared to APAF1 in many cells, with a ratio of ~1:10 and even lower, depending on the cell line [21, 30, 31]. Therefore, our findings suggest that homodimerisation by the induced-proximity model under these conditions is highly unlikely, particularly given reports that only a subset of the PC9 pool binds the apoptosome at any one time [18, 32]. Given this problem, we noted that in recent SPR data a non-cleavable form of PC9 (PC9-TM) appears to bind better to the caspase activation and recruitment domain (CARD) of APAF1 than a non-dimerising PC9 mutant (PC9^F404D^) [16]. It was suggested that this was likely a result of the increased avidity by the collective strength of two CARD_PC9_–CARD_APAF1_ interactions in a homodimer, thus reducing unbinding. However, extraction of the SPR curves (Fig. 2a) and the calculation of the binding (*k*_on_) and unbinding (*k*_off_) kinetics demonstrated that binding was also reduced for non-dimerising PC9^F404D^ compared to PC9-TM (Fig. 2b). Given that at physiological concentrations, cytoplasmic PC9 is maintained as a monomer, we speculated that the initial, relatively unstable recruitment of a single PC9 may facilitate the consequent secondary recruitment of PC9 (Fig. 2c). This cooperative recruitment would then pull PC9 directly into a homodimer potentially increasing homodimerisation efficiency and consequent autocleavage. We implemented this model by performing a parameter estimation using the SPR data as guidelines and previously obtained experimental data on C3-substrate cleavage and the molecular timer activity as training sets (full details in Supplementary Text 2). The consequent cooperative-binding model indeed restored PC9 processing in line with reported turnover rates (Fig. 2d, Supplementary Fig. 1bii) whilst maintaining experimental C3-substrate cleavage rates (Fig. 2e). Moreover, the increased processing of PC9 enabled reproduction of experimental data on the molecular timer effect (Fig. 2f) and XIAP-inhibition of PC3 cleavage (Fig. 2g). Furthermore, flux analysis confirmed that PC9 recruitment was rapidly followed by PC9 cleavage and C9 dissociation (Supplementary Fig. 1aii). This demonstrates that cooperative PC9 recruitment to the apoptosome is essential for rapid PC9 cleavage and consequent regulation of apoptosome activity by both XIAP and the molecular timer.

### The molecular timer operates across a wide-range of physiological protein concentrations

**Fig. 3:**
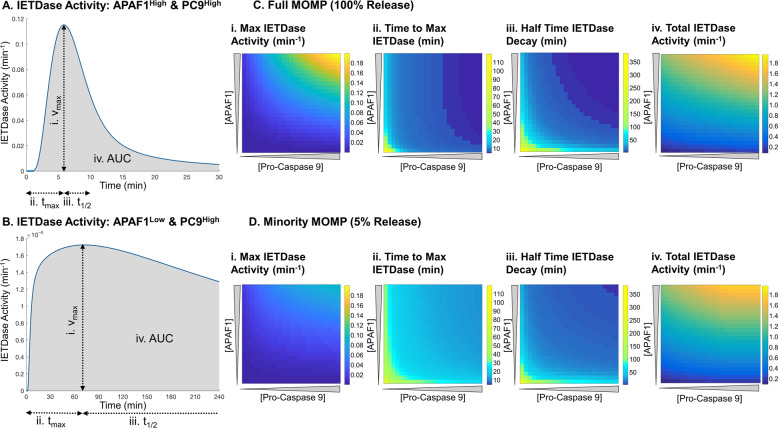
The molecular timer operates at all concentrations of pro-caspase 9 and APAF1. Representative traces of IETDase activity after MOMP with abundant PC9 and APAF1 (**a**) or when one is limiting (**b**). Screening of the maximum IETDase activity (i), time to max IETDase activity (ii), half-time for IETDase decay (iii) and total IETDase activity (iv) after MOMP (**c**) or minimal MOMP (**d**). Half-times were calculated by fitting to one phase decay equation in Matlab. Total IETDase was calculated using the area under the curve.

We next set out to establish if the molecular timer operated at physiological protein levels and under what circumstances is it active. Therefore, we screened previously determined protein ranges of APAF1 and PC9 and calculated the activity of the holo-apoptosome in cleaving the IETD site in PC3 (IETDase) over time [21, 30, 31, 33]. To prevent confounding effects from XIAP activity, protein synthesis and degradation, these factors were excluded from the screening. Across all conditions tested the IETDase activity reached a maximum activity within 2 h before declining, indicative of the molecular timer, even at very low levels of PC9 or APAF1 (data not shown). However, the profile of IETDase activity was markedly different depending on the levels of PC9 and APAF1. When both PC9 and APAF1 were abundant (representative curve Fig. 3a), the IETDase activity rapidly shoots up and is almost equally as rapidly shut down again to give a strong short pulse of activity. On the other hand, when one or both of PC9 or APAF1 were low the activity was much reduced but shut down slower (representative curve Fig. 3b).

To characterise the concentration-dependent effects of PC9 and APAF1 on the molecular timer effect better, we calculated the maximum IETDase activity, time to maximum IETDase activity and half time of IETDase decay across 4 h for every condition (Fig. 3a, b). This confirmed that at high abundance of PC9 and APAF1, strong IETDase activity is induced rapidly (Fig. 3ci–ii) before its rapid decline (Fig. 3ciii). However, when PC9 or APAF1 are low, the maximum activity is reduced but the decline in activity is much slower. We postulated that this may act as an intrinsic mechanism of dosage compensation to ensure that a significant level of apoptosome activity always occurs before molecular timer mediated shutdown. To investigate this hypothesis, we calculated the total IETDase activity across 4 h of simulation using the AUC of the IETDase traces. This confirmed that even with low maximal IETDase activity (Fig. 3ci), the total IETDase activity is partially offset by the slow onset and shutdown of the IETDase activity (Fig. 3civ).

The molecular timer was originally proposed as a mechanism to prevent apoptosis under circumstances of accidental low-level release of apoptotic factors [18]. Therefore, we further investigated whether the molecular timer is indeed operational under such a scenario by simulating minMOMP, defined as release of 5% of CytC (Fig. 3d) [19]. Similar to full MOMP, the molecular timer was active under all combinations of PC9 and APAF1. Generally, maximum activity was reduced, with a slower onset and slower degradation compared to full MOMP (Fig. 3di–iii). This is likely attributable to the delay and reduced efficiency in apoptosome formation with sub-maximal release of CytC (Supplementary Fig. 2). However, once more an intrinsic dosage compensation was observed in total IETDase activity to offset the lower maximal IETDase activity (Fig. 3div). These results demonstrate that the molecular timer operates across a range of physiological APAF1 and PC9 concentrations after both complete and minMOMP.

### The apoptosome molecular timer can prevent apoptosis execution

**Fig. 4:**
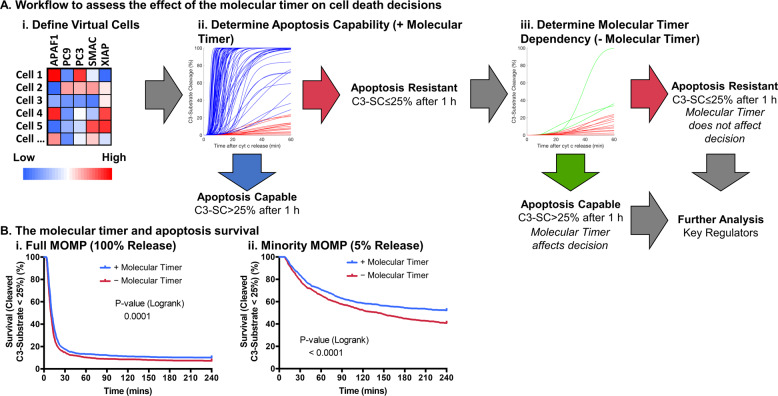
Mathematical modelling suggests that the molecular timer can prevent apoptosis. A workflow for assessment of the molecular timer contribution to apoptosis prevention (**a**). Virtual cells were defined from physiological protein levels (i) to be tested for apoptosis resistance (ii). Apoptosis resistant cells are then simulated without the molecular timer (iii) to look for molecular timer dependent resistance to apoptosis. Survival curves with and without the molecular timer (**b**) for complete MOMP (i) and minority MOMP (ii). Apoptosis death event was considered to be when 25% of C3-substrate cleavage is reached. *P* values in B from logrank test, *n* = 1000 for all groups.

Our previous results indicate that the molecular timer is active under physiological conditions. However, it remains unknown so far if the molecular timer can prevent lethal caspase 3 activation and apoptosis execution. Moreover, determining the contribution of the molecular timer to apoptosis resistance in intact cells experimentally cannot be separated from coupled processes such as XIAP-inhibition and C3-feedback cleavage of C9 and XIAP [17, 34]. Mathematical modelling allows the facile uncoupling of such processes enabling elucidation of their contribution to complex system outcomes. Therefore, in order to determine if apoptosis execution could be prevented by the molecular timer we sampled physiological protein ranges to define 1000 virtual cells and tested if they were apoptosis capable or resistant (Fig. 4ai–ii). Apoptosis resistance was defined as less than 25% C3-substrate cleavage, in line with previous experimental studies where all cells with less than 25% cleavage of a FRET-based C3-substrate showed no morphological signs of apoptosis [21]. Conditions that demonstrated apoptosis resistance were then further analysed for whether removal of the apoptosome molecular timer sensitised to apoptosis execution or not (Fig. 4aiii).

The molecular timer could prevent apoptosis only in a small subset of cases after full MOMP (Fig. 4bi). In contrast, for conditions of minMOMP [19], the role of the molecular timer in preventing apoptosis execution was considerably more pronounced, rescuing approximately one in four cells that otherwise would respond with apoptosis execution (Fig. 4bii). A similar pattern was also observed when using a less stringent cut-off for apoptosis resistance of 80% C3-substrate cleavage (Supplementary Fig. 4). Correspondingly, performing these analyses in presence of non-cleavable PC9, a molecular tool used for experimentally studying the absence of the molecular timer but which also cannot be inhibited by XIAP, resulted in increased apoptosis susceptibility compared to removal of the molecular timer alone (Supplementary Fig. 5). Together, these results suggest that the molecular timer can prevent apoptosis execution in a subset of physiological conditions, and particularly prominently at conditions of minMOMP.

### The apoptosome molecular timer’s ability to prevent apoptosis is intricately linked to XIAP-SMAC balance

**Fig. 5:**
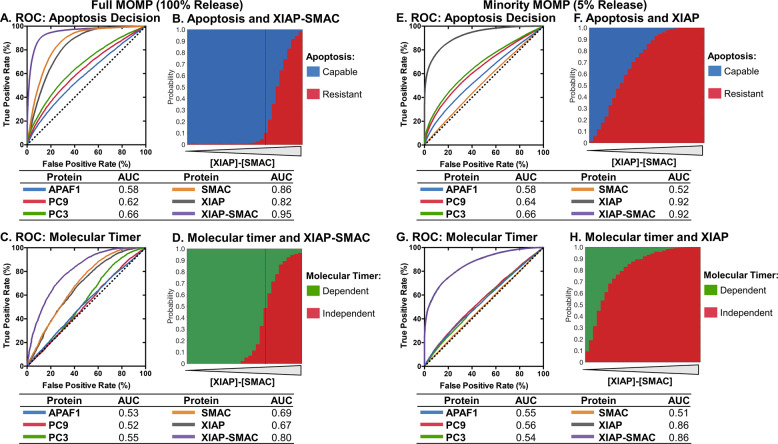
Mathematical modelling demonstrates that the molecular timer’s ability to prevent apoptosis is intricately linked to XIAP-SMAC balance. ROC and AUC of the ability of apoptosis proteins to affect apoptosis decisions after MOMP (**a**). XIAP-SMAC concentration balance and apoptotic decisions after MOMP (**b**). ROC and AUC of the ability of apoptosis proteins to affect molecular timer dependency of apoptotic resistance after MOMP (**c**). XIAP-SMAC concentration balance and the molecular timer dependency of apoptotic resistance after MOMP (**d**). ROC and AUC of the ability of apoptosis proteins to affect apoptosis decisions after minimal MOMP (**e**). XIAP concentration and apoptotic decisions after minimal MOMP (**f**). ROC and AUC of the ability of apoptosis proteins to affect molecular timer dependency of apoptotic resistance after minimal MOMP (**g**). XIAP concentration and the molecular timer dependency of apoptotic resistance after minimal MOMP (**h**). Dotted lines in B and D indicates the point where XIAP and SMAC are equimolar. Dotted lines in A, C, E and F indicates identity line where false positive rate equals true positive rate. Data based on 100000 simulations for MOMP and minimal MOMP.

We next aimed to identify the key determiners of conditions under which the molecular timer is relevant for preventing apoptosis execution. To quantify the individual relevance of each key protein involved in apoptosis execution, we employed the receiver operator curve (ROC) and the AUC for evaluating the ability to predict apoptosis competency and molecular timer dependence. As expected, low levels of pro-apoptotic proteins APAF1, PC9, PC3 and SMAC can mediate apoptosis resistance, with SMAC having a particularly strong influence (AUC = 0.86) (Fig. 5a and Supplementary Fig. 6). Likewise, high levels of XIAP correlated with apoptosis resistance (AUC = 0.82), in line with previous findings [35–37]. As XIAP is inhibited by SMAC, we also looked at how the balance of XIAP and SMAC affected apoptosis resistance. Strikingly, the balance of XIAP and SMAC strongly influenced apoptosis decisions (AUC = 0.95) showing an almost binary separation at the point XIAP and SMAC are equimolar (Fig. 5a, b and Supplementary Fig. 6). XIAP and SMAC were also the main determinants for whether the apoptosome timer was relevant for preventing apoptosis execution, achieving an AUC of 0.80 for the XIAP-Smac balance (Fig. 5c and Supplementary Fig. 7). Notably, when XIAP exceeds SMAC, the molecular timer has little influence on cell death decisions, whereas for excess SMAC the molecular timer is a key factor for preventing apoptosis (Fig. 5d). We interpret this outcome as the molecular timer playing its most prominent role when apoptosis resistance is not completely dictated by XIAP. Interestingly, the proteins involved in the timer itself, APAF1 and PC9, had little influence on apoptosis decisions, likely reflecting our finding that the molecular timer is active under all conditions and has its own intrinsic dosage compensation mechanism (Fig. 3).

For conditions of minMOMP, XIAP was identified as the major determiner of apoptosis resistance (AUC = 0.92) (Fig. 5e and Supplementary Fig. 8). The limited release of SMAC under these conditions limits its contribution and consequently the XIAP-SMAC balance does not improve the classification (Fig. 5e, f). XIAP also had the most influence on the molecular timer’s ability to prevent apoptosis (Fig. 5g, h and Supplementary Fig. 9).

Taken together, these results demonstrate that the molecular timer’s ability to prevent apoptosis is complementary to XIAP, with the timer gaining relevance at conditions of low XIAP activity but also actively preventing apoptosis even when XIAP is in excess.

### The molecular timer acts synergistically with XIAP in order to shutdown apoptosis

**Fig. 6:**
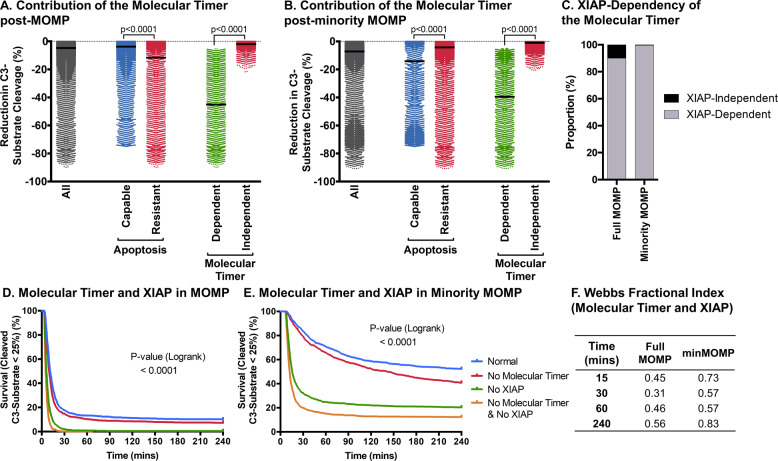
The molecular timer acts synergistically with XIAP in order to shut down the apoptosome. The contribution of the molecular timer, calculated as the difference in C3-substrate cleavage with and without the molecular timer, 1 h post-MOMP (**a**) or post-minority MOMP (**b**). The proportion of molecular timer dependent apoptosis resistant cells that are no longer resistant following removal of XIAP (**c**). Survival curves under normal conditions, without XIAP, without the molecular timer or without XIAP and molecular timer after MOMP (**d**) or minority MOMP (**e**). Synergy scores for XIAP and the molecular timer calculated using Webb’s Fractional Index at different time intervals after MOMP or minority MOMP (**f**). Data in **a**–**c** based on 100,000 simulations for MOMP and minimal MOMP. *P* values in **a** by Kruskal–Wallis with Dunn’s correction. *P* values in D and E were obtained by logrank test, *n* = 1000 for all groups.

Since PC9 autocatalytic cleavage initiates the roles of both the molecular timer and XIAP in apoptosis execution, we explored the relationship of both further. We found that the molecular timer reduced C3-substrate cleavage under all conditions, after full MOMP (Fig. 6a) or minMOMP (Fig. 6b). This demonstrates that even when the molecular timer alone cannot prevent apoptosis, it still contributes substantially to apoptosis shutdown by XIAP. Correspondingly, when we removed XIAP from conditions where apoptosis resistance was molecular timer dependent, such cells now became apoptosis capable (Fig. 6c). Therefore, the molecular timer almost exclusively depends on cooperation with XIAP in order to prevent apoptosis. To observe their separate and combined contributions, we studied C3-substrate cleavage in the absence of one or both for conditions of MOMP (Fig. 6d) or minMOMP (Fig. 6e). The removal of both XIAP and the molecular timer decreased apoptosis resistance more than the removal of one or the other. This difference was particularly pronounced after minMOMP. Next, we looked to ascertain if the molecular timer and XIAP’s ability to prevent apoptosis is additive or shows inherent synergism (Fig. 6f). This indeed demonstrated that XIAP and the molecular timer operate synergistically in the prevention of apoptosis after MOMP and minMOMP. Applied to HeLa cells undergoing minMOMP conditions, as reported previously [19], very little active C3 formed after partial release of SMAC and CytC, which was reversed by removal of either XIAP or the molecular timer (Supplementary Fig. 10), demonstrating that the molecular timer promotes survival of HeLa cells after minMOMP. This demonstrates that PC9 autocatalytic cleavage triggers XIAP and the molecular timer to synergistically prevent apoptosis after MOMP and minMOMP.

### Mathematical modelling prognosticates treatment response and demonstrates a role for the molecular timer in patients with stage III colorectal cancer

**Fig. 7:**
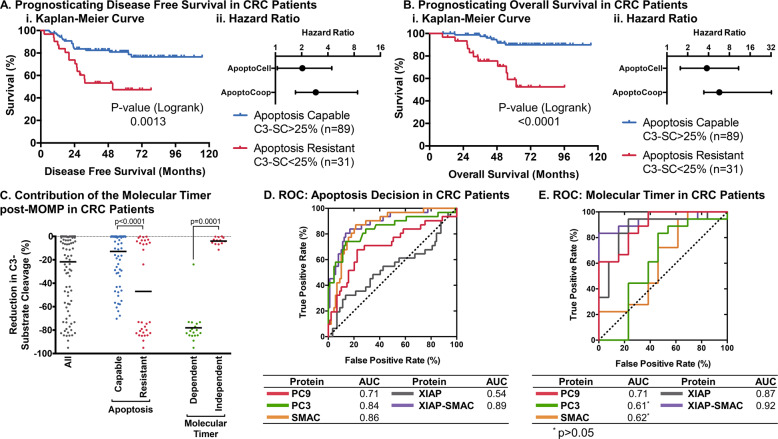
The molecular timer may contribute to treatment resistance in patients with stage III colorectal cancer. The cooperative recruitment model (ApoptoCoop) was used to classify 120 patients with stage III colorectal as apoptosis capable or resistant by C3-substrate cleavage after 300 min and was used to draw Kaplan–Meier curves (i) of disease free survival (**a**) and overall survival (**b**) and compare hazard ratios against a simplified model of apoptosis (ApoptoCell) (ii). The contribution of the molecular timer, calculated as the difference in C3-substrate cleavage with or without the molecular timer, 5 h post-MOMP in colorectal patients (**c**). ROC and AUC of the ability of apoptosis proteins to affect apoptosis decisions after MOMP in colorectal cancer patients (**d**). ROC and AUC of the ability of apoptosis proteins to affect molecular timer dependency of apoptotic resistance after MOMP in colorectal cancer patients (**e**). *P* values in C by Kruskal–Wallis with Dunn’s correction.

Previously, we demonstrated that a simplified model of apoptosis execution was prognostic for disease free and overall survival in stage III colorectal cancer patients treated with chemotherapy [24]. We therefore aimed to discover if the molecular timer can contribute to correctly prognosticating patient outcome. Protein concentrations for PC9, PC3, XIAP and SMAC from *n* = 120 patient tumours, quantified by reverse phase protein array [24], together with a median APAF1 expression [25] were used to simulate apoptosis execution. Patients were then separated into groups bearing tumours expected to be apoptosis susceptible (calculated C3-substrate cleavage >25%) and apoptosis resistant (C3-substrate cleavage ≤25%). Patients with apoptosis capable tumours demonstrated an increased disease free (Fig. 7ai) and overall survival (Fig. 7bi). Our model outperformed the previous simplistic execution phase model as evidenced by the increased hazard ratio (Fig. 7a, bii). This demonstrates that the more detailed modelling of events at the apoptosome such as the molecular timer improves systems-based prognosis based on apoptosis execution proteins.

We next looked at the relative contribution of the molecular timer in this patient cohort. 18/31 (58%) of apoptosis resistant tumours were resistant due to the apoptosome molecular timer, suggesting that the molecular timer indeed contributes substantially to apoptosis resistance in cancer patients. Moreover, the reduction in C3-substrate cleavage attributable to the molecular timer demonstrated a very strong reduction of >70% in 17/18 of patients that were resistant due to the molecular timer (Fig. 7c). Varying APAF1 concentrations affected classification of patients only in two cases at extremely low levels of APAF1 (data not shown), reciprocating our previous findings that APAF1 has only a minor influence on apoptosis competency.

Analysing the contribution of each individual protein to apoptosis decisions interestingly identified PC9 and PC3 to strongly contribute to correctly classifying apoptosis competency in colorectal cancer (Fig. 7d). Moreover, the relative influence of XIAP (AUC = 0.54) was much reduced when compared to our unbiased screen across protein expression ranges (Fig. 5), whilst its inhibitor SMAC had the strongest effect on apoptosis decisions (AUC = 0.86). The balance of XIAP and SMAC best-predicted apoptosis competency. The latter reinforces our previous finding, with the relatively small contribution of XIAP alone in the colorectal cohort likely due to its relatively low expression variance within the cohort itself (Coefficient of variation for XIAP compared to PC9, PC3 and SMAC = 100% vs 154%, 144% and 135%, respectively). Next, we looked at key markers of the molecular timer’s ability to mediate apoptosis resistance. Despite its seemingly reduced contribution to apoptosis decisions in colorectal cancer, XIAP was still the best singular protein in determining the relative influence of the molecular timer (Fig. 7e). In line with our above findings, XIAP and SMAC balance was the best predictor for the molecular timer’s ability to mediate apoptosis resistance. In summary, we show that considering the apoptosome molecular timer notably improves apoptosis competency-linked prognosis of CRC stage III patient outcome.

## Discussion

In this paper, we demonstrate that the apoptosome molecular timer acts synergistically with XIAP in the prevention of apoptosis execution and that simulating these processes can contribute to correctly prognosticate patient survival in stage III colorectal cancer. Apoptosome activity is dictated by PC9 recruitment, activation and cleavage on the apoptosome. These processes are highly complex to study experimentally and therefore are not fully understood. We employed a systems approach to demonstrate that the induced-proximity dimerisation model of PC9 homodimerisation is insufficient to produce efficient autocatalytic cleavage of PC9. Previously, it has been demonstrated using non-cleavable mutants of PC9 that this step is not necessary for its ability to cleave PC3 [38]. However, the inhibitor XIAP requires the short ATPFQEG peptide sequence on C9, exposed by D^315^ autocatalytic cleavage, to have sufficient affinity to bind and block C9 activity [9, 17, 18]. Furthermore, non-cleavable PC9 mutants appear to have a much-increased activity in cell-free reconstituted apoptosome and cell extract experiments, independent of the role of XIAP, due to the low affinity of C9-35/12 to the apoptosome [16, 18]. Therefore, the efficient autocleavage of PC9, as exhibited by our cooperative binding model, is a key feature in negative regulation of apoptosis execution. The SPR experiment, on which this model is based, used mutant and purified proteins [16]. We cannot therefore rule out the possibility of artefacts caused by using such mutants or proteins purified to higher than physiological concentrations. Indeed, overexpression of PC9 can lead to spontaneous dimerisation of a subset of PC9, that is not readily observed at the physiological levels found in cells [16]. However, our reported stochiometric ratio of PC9 to APAF1 would suggest that such a cooperative C9 recruitment would exist given the rapid cleavage of PC9 [26–29]. PC9 appears to be maintained in a monomeric form outside the apoptosome by its own CARD inhibiting dimerisation, with the CARD^PC9^-CARD^APAF1^ interactions at the apoptosome inducing a conformational change removing this apparent auto-inhibition to allow PC9 dimerisation and activity [39]. Cooperative recruitment could help induce and stabilise the necessary conformational changes for binding by the exposed dimerisation domain of a bound PC9 actively competing with the CARD of unbound autoinhibited PC9 for its dimerisation domain. The cooperative recruitment of C9 to the apoptosome has thus far not been reported, however cooperativity has previously been implicated in the recruitment of proteins to the death-inducing signalling complex in extrinsic apoptosis as well as for other proteins with multiple binding sites and in binding of transcription factors to DNA [40–43].

Implementing cooperative binding of C9 to the apoptosome results in excellent agreement between simulation results and a multitude of previous experimental data, and allowed us to confidently screen for biological phenomena. Furthermore, we demonstrated that our model was prognostic for both disease free and overall survival in stage III colorectal cancer by separating patients into apoptotic resistant and apoptotic capable groups using C3-substrate cleavage levels. Apoptosis resistance has been implicated in both treatment resistance and tumour progression [44–46], therefore providing a logical link between apoptosis execution and patient outcomes. Interestingly, the current version of our model outperformed a simplified model of apoptosis execution that contains many of the same reactants and reactions as implemented in this paper but greatly simplified complex events at the apoptosome, using a PC9 activation kinetic derived from APAF1 and PC9 levels [21, 47]. Notably, the improvement in prognostic capacity was mainly derived from reclassification of patients to apoptosis capable. A number of factors may explain this reclassification. Firstly, ~15% of entire apoptosome activity is mediated by activation of PC9 [18] and therefore is not blocked by XIAP, a feature replicated in our cooperative binding model (see Fig. 2g) but not in previously published models. This activity will also increase the amount of XIAP required to achieve effective inhibition as PC3 levels often are in excess to PC9 levels (see Fig. 4a**i**). The improvement in prognostic ability of our model therefore indicates that more realistic modelling of complex events is important in fully understanding patient treatment responses and prognosis.

The molecular timer, whilst an interesting effect, has yet to be conclusively demonstrated to operate in real cells and to prevent apoptosis experimentally. Previous studies have used non-cleavable forms of PC9 in order to show the molecular timer effect [16, 18]. However, we have demonstrated that the molecular timer’s ability to prevent apoptosis is intricately linked to XIAP, which is also unable to block non-cleavable PC9 [9, 17, 18]. Indeed, simulating survival of cells using non-cleavable PC9 led to increased apoptosis compared to simply turning the molecular timer off (Supplementary Fig. 5). Therefore, delineating when the molecular timer is able to prevent apoptosis is highly complex experimentally. We here have employed a screen using our validated model, which demonstrates that the molecular timer may actively prevent apoptosis execution. Previously, it was hypothesised in the work of Malladi et al. that this would protect against the accidental partial release of apoptotic factors, which is in line with our findings for simulated minMOMP [18]. However, we also showed that it could protect in a subset of scenarios after complete MOMP, including in simulations utilising protein levels seen in patients with stage III colorectal cancer. This suggests that this mechanism can be employed as a more universal strategy to prevent apoptosis. Moreover, whilst not included in our simulations, sufficient activation of C3 leads to D^330^ feedback cleavage of C9, which when combined with D^315^ autocatalytic cleavage to form C9-35/10 fully removes the p2 fragment containing the high affinity XIAP-binding domain creating a positive feedback loop [17]. Intriguingly, biochemical studies have suggested that the dual cleaved C9-35/10 form has more activity than either of the single cleaved isoforms, C9-35/12 or C9-37/10 [16]. It was concluded that this was due to partially restored affinity of C9-35/10 to the apoptosome. This would create an elegant switch mechanism for tandem prevention of apoptosis by both XIAP and the molecular timer, initiated by D^315^ cleavage and terminated by D^330^ feedback cleavage in the event of sufficient C3 activation. There are several plausible hypotheses for why such a mechanism would exist, which are not necessarily mutually exclusive. First, non-lethal and low-level activation of C3 has been demonstrated to have pro-tumourigenic effects, promoting DNA-instability and pro-survival responses such as NFκB [19, 48]. Second, when apoptosis execution is blocked after MOMP, cells still usually undergo cell death in a caspase-independent manner also associated with an inflammatory response [49–51]. The best characterised mechanism for this is release of mitochondrial DNA and triggering of a Gas-Sting-Interferon response that is actively inhibited by caspase 3-mediated cleavage of Gas and interferon regulatory factor 3 (IRF3) [52–56]. Therefore, shutting down of apoptosis execution signalling by tandem XIAP-inhibition and the molecular timer can prevent low-level non-lethal caspase 3 activity that may promote tumorigenesis and block inflammatory responses post-MOMP. Activation of such an inflammatory cell death has been mooted as a potential mechanism to improve anti-cancer therapy by killing cancerous cells and activating the immune system in a single stroke [19, 57, 58]. However, we observed a poorer outlook for apoptotic resistant cells suggesting that such a mechanism is not always beneficiary or may also be concurrently blocked. Indeed, perturbations of the Gas-Sting axis was frequently observed in a panel of colorectal cancer cell lines and stage II/III colorectal patients [59, 60].

In conclusion, we have utilised a novel system biology approach to demonstrate that cooperative recruitment of PC9 to the apoptosome is a key process in regulation of apoptosis by both XIAP and the molecular timer. Moreover, employing this model in screening suggests that the molecular timer can actively prevent apoptosis after minMOMP and MOMP in physiological settings. Finally, we showed that our model has prognostic capability and indicates that the molecular timer can contribute to patient outcomes.

## Supplementary information

Supplementary Material

## Data Availability

All raw data and processing scripts are available from the corresponding author upon reasonable request.

## Code availability

The model has been uploaded to the data repository Zenodo under a Creative Commons Attribution 4.0 International license (10.5281/zenodo.3712773) along with setup files to replicate figures within this paper. Scripts for processing of patient data was previously made available on Zenodo (10.5281/zenodo.1162682). All other codes are available on reasonable request from the corresponding author.
