# Supplementary material for: The apoptosome molecular timer synergises with XIAP to suppress apoptosis execution and contributes to prognosticating survival in colorectal cancer
Source: Cell Death Differ. 2020 Apr 27;27(10):2828–42. doi: 10.1038/s41418-020-0545-9 (PMC7493894; doi:10.1038/s41418-020-0545-9)
Supplement: Supplementary file 1 — Supplementary Material [file 41418_2020_545_MOESM1_ESM.pdf]

Supplementary Materials for 'The apoptosome molecular timer synergises with XIAP to suppress apoptosis execution and contributes to prognosticating survival in colorectal cancer'

Table of Contents

Supplementary Text 1: Parameterisation of models of the apoptosis execution phase............1

Table 1: List of starting concentrations in HeLa cells............2

Table 2: List of molecules not present at the start of the simulation (initial concentration = 0 µM).......3

Table 3: List of key reactions and their descriptions............4

Table 4: List of reactions, forward and reverse rates in the original ApoptoAll model............5

Table 5: List of protein degradation reactions and their kinetic rates in the models............6

Table 6: Differences in reactions and reaction rates between Apopto-All, -DimC and -Coop............6

Figure 1: Turning off the molecular timer............7

Supplementary Text 2: Parameter Estimation............7

Table 1: Upper and lower bounds used for the parameter estimation............8

Supplementary Figures:............9

Supplementary Figure 1............9

Supplementary Figure 2............10

Supplementary Figure 3............10

Supplementary Figure 4............11

Supplementary Figure 5............11

Supplementary Figure 6............12

Supplementary Figure 7............12

Supplementary Figure 8............13

Supplementary Figure 9............13

Supplementary Figure 10............14

Supplementary Text 1: Parameterisation of models of the apoptosis execution phase

Kinetic values are based on the previously published model of allosteric and homodimerisation mediated cleavage at the apoptosome (ApoptoAll). All kinetic values in the ApoptoAll model are based on experimental data. Kinetic values for apoptosome formation were determined by a kinetic screening for K_D, k_{on} and k_{off} values that allowed for 65% apoptosome formation within 5

min after CytC release in line with experimental data (1). Kinetic rates for the binding of PC9 and C9 to the apoptosome were determined by a kinetic screening of k_{on} and k_{off} value pairs to a experimentally determined K_D value (2,3). All models require initial concentrations of PC3, PC9, Apaf-1, XIAP, mitochondrial Smac, mitochondrial cyt c and ATP (Table 1). Figure 1A, C & D and Figure 2D, E & H were based on HeLa cell quantification of these proteins, as reported previously (3,4). In parameter screens, cytochrome C (5) and ATP (6) levels were taken from HeLa cells as generally they are not thought to be rate limiting.

Table 1: List of starting concentrations in HeLa cells

Name	Description	HeLa
PC3	Procaspase-3	0.12
PC9	Procaspase-9	0.03
Apaf-1	Apaf-1	0.372
XIAP	XIAP	0.063
$Smac_{mito}$	Mitochondrial Smac	0.126
$CytC_{mito}$	Mitochondrial Cytochrome C	10
ATP	ATP	920

Species that are not present at the start of the simulation, but are formed during apoptosis execution, are displayed in Table 2. The key reactions and kinetic parameters of the models are further summarised in Table 3 and Table 4. Most species are additionally modelled to undergo degradation with a specific degradation rate (Table 5). Additionally, Apaf-1, PC9, PC3 and XIAP are also formed *de novo* in the models. The formation rate is calculated by multiplying the kinetic rate of the degradation reaction of each protein with its initial concentration.

Table 2: List of molecules not present at the start of the simulation (initial concentration = 0 μM).

Protein	Description
C3	Caspase-3
C9	Caspase-9
CytC	Cytosolic Cytochrome C
SMAC	Cytosolic SMAC
[APAF1~CytC]	Monomeric Apaf-1 bound to CytC
[APAF1~ATP]	Monomeric Apaf-1 bound to ATP
[APAF1~CytC~ATP]_n	Apaf-1 oligomer, where n can be any number between 1 and 6 representing a Apaf-1 monomer, dimer, trimer, tetramer, pentamer or hexamer respectively
Apoptosome	Fully assembled heptameric Apaf-1 complex
[Apoptosome_PC9_n_C9_m]	Fully assembled heptameric Apaf-1 complex with bound PC9 and/or C9. Both n and m can be any number between 0 and 7, however $n + m \leq 7$.
[Apoptosome_P9_n_C9_m~XIAP]	Apoptosome complex with bound XIAP. n can be any number between 0 and 7, m any number between 1 and 7, however $n + m \leq 7$.
[XIAP~SMAC]	XIAP bound to Smac
[XIAP~C3]	XIAP bound to Caspase-3
Substrate_C3	Caspase-3 substrate
clSubstrate_C3	Cleaved Caspase-3 Substrate

Table 3: List of key reactions and their descriptions.

#	Reaction	Description
1	CytC_Mito => CytC	Release of CytC from the mitochondria.
2	SMACmito => SMAC	Release of Smac from the mitochondria.
3	APAF-1 + ATP <=> [APAF1~ATP]	Binding of ATP to Apaf-1.
4	APAF-1 + CytC <=> [APAF1~CytC]	Binding of CytC to Apaf-1.
5	[APAF1~ATP] + CytC <=> [APAF1~CytC~ATP]	Formation of active state of Apaf-1.
6	[APAF1~CytC] + ATP <=> [APAF1~CytC~ATP]	Formation of active state of Apaf-1.
7	[APAF1~CytC~ATP]_n + [APAF1~CytC~ATP]_m <=> [APAF1~CytC~ATP]_k	Formation of Apaf-1 oligomers. $n + m \leq 6, k \leq 6$
8	[APAF1~CytC~ATP]_n + [APAF1~CytC~ATP]_m => Apoptosome	Formation of the apoptosome. $n + m = 7$
9	[Apoptosome_PC9_n_C9_m] + PC9 <=> [Apoptosome_PC9_n+1_C9_m]	Binding of PC9 to the Apoptosome. $n + m \leq 6$
10	[Apoptosome_PC9_n_C9_m] + C9 <=> [Apoptosome_PC9_n_C9_m+1]	Binding of C9 to the Apoptosome. $n + m \leq 6$
11	[Apoptosome_PC9_n_C9_m] => [Apoptosome_PC9_n-1_C9_m+1]	Autocatalytic cleavage of PC9 monomers. $n = 1$
12	[Apoptosome_PC9_n_C9_m] => [Apoptosome_PC9_n-1_C9_m+1]	Autocatalytic cleavage of PC9 dimers. $n \geq 2$
13	[Apoptosome_PC9_n_C9_m] => [Apoptosome_PC9_n-2_C9_m+2]	Autocatalytic cleavage of PC9 dimers. $n \geq 2$
14	[Apoptosome_PC9_n_C9_m] + PC9 => [Apoptosome_PC9_n+1_C9_m-1] + C9	Substitution of C9 by PC9. $m \geq 1$
15	[Apoptosome_PC9_n_C9_m] + PC3 => [Apoptosome_PC9_n_C9_m] + C3	C3 cleavage mediated by PC9. $n > m$
16	[Apoptosome_PC9_n_C9_m] + PC3 => [Apoptosome_PC9_1_C9_1] + C3	C3 cleavage mediated by C9. $n \leq m$
17	C3 + Substrate_C3 => C3 + clSubstrate_C3	C3 substrate cleavage by C3
18	[Apoptosome_PC9_n_C9_m] + C3 => [Apoptosome_PC9_n-1_C9_m+1] + C3	Feedback cleavage of C3 on apoptosome-bound PC9. $n \geq 1$
19	PC9 + C3 => C9 + C3	Feedback cleavage of C3 on cytosolic PC9.
20	[Apoptosome_PC9_n_C9_m] + XIAP <=> [Apoptosome_PC9_n_C9_m~XIAP]	Binding of XIAP to the apoptosome. $m \geq 1$
21	XIAP + C3 <=> [XIAP~C3]	Binding of XIAP to C3.
22	XIAP + SMAC <=> [XIAP~SMAC]	Binding of Smac to XIAP.
23	[Apoptosome_PC9_n_C9_m~XIAP] + SMAC <=> [Apoptosome_PC9_n_C9_m] + [XIAP~SMAC]	Binding of Smac to apoptosome-bound XIAP. $m \geq 1$
24	[XIAP~C3] + SMAC <=> [XIAP~SMAC] + C3	Binding of Smac to C3-bound XIAP.

$n + m \leq 7$, unless indicated otherwise.

Table 4: List of reactions, forward and reverse rates in the original ApoptoAll model.

#	Forward rate [μM^{-1} min^{-1}]	Reverse rate [min^{-1}]	Reference
1	0.4621		(5,13)
2	0.099		(4)
3	0.1359	0.1155	(14,15)
4	0.24	0.006	(16,17)
5	0.24	0.006	K_D(16), k_{off}(17)
6	0.1359	0.1155	(14,15)
7	40	0.004	(18)
8	40		(18)
9	2.85	2	(2,8)
10	0.285	2	(2,8)
11	73.38		k_{cat}(19), K_m(8)
12	73.38		k_{cat}(19), K_m(8)
13	73.38		k_{cat}(19), K_m(8)
14	2.85		(2,8)
15	63.38		k_{cat}(19), K_m(8)
16	73.38		k_{cat}(19), K_m(8)
17	12		(20)
18	0.105		(21)
19	0.105		(21)
20	156	0.144	(22)
21	156	0.144	(22)
22	420	0.133	(23)
23	420	156	(23)
24	420	156	(23)

Table 5: List of protein degradation reactions and their kinetic rates in the models.

ID	Reaction	Forward rate [min^{-1}]	Reference
24	APAF-1 =>	0.00048	(3)
25	CytC =>	0.000385	(24)
26	[APAF1~ATP] =>	0.0058	(25)
27	[APAF1~CytC] =>	0.0058	(25)
28	[APAF1~CytC~ATP]_n =>	0.0058	(25)
29	[Apoptosome_PC9_n_C9_m] =>	0.0039	(25)
30	[Apoptosome_PC9_n_C9_m~XIAP] =>	0.0039	(25)
31	PC3 =>	0.00048	(25)
32	PC9 =>	0.00048	(25)
33	C3 =>	0.0058	(25)
34	C9 =>	0.0058	(25)
35	SMAC =>	0.0058	(24)
36	[XIAP~C3] =>	0.0347	(26)
37	[XIAP~SMAC] =>	0.0347	(26)
38	XIAP =>	0.0116	(25)

The models with only homodimerisation-mediated cleavage (ApoptoDimC) and cooperative recruitment (ApoptoCoop) models are based on the original ApoptoAll model and the differences between the models are highlighted in Table 6.

Table 6: Differences in reactions and reaction rates between Apopto-All, -DimC and -Coop

Reaction	ApoptoAll	ApoptoDimC	ApoptoCoop	
Binding of PC9 to Apoptosome occupied with only C9	✕	✓	✓	
Autocatalytic cleavage of PC9 monomers.	✓	✕	✕	
Substitution of C9 by PC9	✓	✓	✕	
C3 feedback cleavage on PC9	✓	✓	✕	
Initial binding of PC9 to the apoptosome	2.85	2.85	1.843	k_{on} [µM^{-1} min^{-1}]
	2	2	1.771	k_{off} [min^{-1}]
Subsequent binding of PC9 to the apoptosome	2.85	2.85	272.225	k_{on} [µM^{-1} min^{-1}]
	2	2	1.203	k_{off} [min^{-1}]
Binding of C9 to the apoptosome	0.285	0.285	0.0049	k_{on} [µM^{-1} min^{-1}]
	2	2	1.994	k_{off} [min^{-1}]
PC9 mediated cleavage of C3	63.38	63.38	20	k_{on} [µM^{-1} min^{-1}]

In all the models used, PC9 and C9 were modelled to only bind to the fully assembled apoptosome, not to any precursor molecules. In the originally published ApoptoAll model(3), C3

was implemented to cleave PC9 to C9. This reaction was turned off in all simulations, since C3 was shown to cleave both PC9 and C9, leading to the formation of new C9 species with different binding behaviour and activity that is not included in the models in this paper(7). Moreover, in the ApoptoAll model, C3-cleavage of PC9 to C9 had no notable effect due to the more rapid monomeric autocleavage of PC9 to C9 (data not shown). Additionally, the activity of PC9 was lowered in ApoptoCoop compared to the allosteric model. This was to reflect the fact that PC9 is not constitutively activated on the apoptosome but likely alternates between active heterodimer and inactive states. Although C9 also is activated as a heterodimer, C9's ability to dimerise is impeded after autocatalytic cleavage, moreover C9-35/12 has poor affinity for the apoptosome and is prone to falling off. Therefore, the majority of C9 activity likely comes from the original PC9 homodimer formed prior to cleavage(7,8). As a further validation of this effect, simulations matched experimental findings that C9-35/12 contributed approximately 85% of PC3 cleavage, as determined using the C9-35/12 specific inhibitor Bir3-RING derived from truncation of XIAP (data not shown)(8).

The model without the molecular timer (ApoptoCoop^{-Timer}) was implemented as ApoptoCoop with the following modification. The k_{on} and k_{off} of C9 binding to the apoptosome were set to the same as secondary binding of PC9, effectively preventing the molecular timer. This was validated by monitoring the IETDase activity with this alteration (ApoptoCoop^{-Timer}), where the activity is both increased and sustained compared to ApoptoCoop, demonstrating the lack of molecular timer activity.

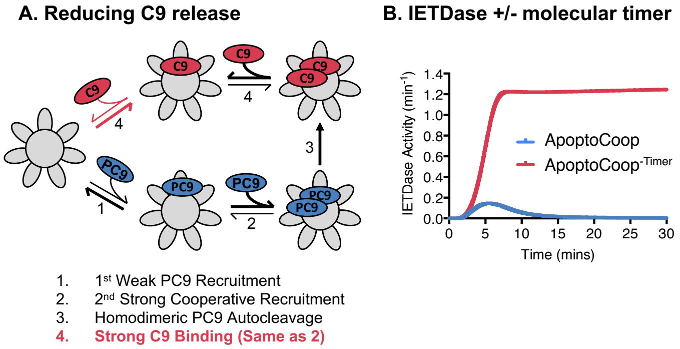

Figure 1: Turning off the molecular timer. Schematic of ApoptoCoop^{-Timer} with reaction 4 (red) altered to prevent the molecular timer (**A**). IETDase activity over time with ApoptoCoop and ApoptoCoop^{-Timer} (**B**). Simulations based on apoptosome activity in HeLa cells.

Supplementary Text 2: Parameter Estimation

In order to implement cooperative recruitment, a parameter estimation was performed to determine kinetic values for primary binding of PC9, cooperative binding of PC9 and binding of C9 to the apoptosome. The upper and lower bounds used for the parameter estimation were chosen with regard to the SPR data (Fig 2) and the original kinetic values from the ApoptoAll

model (Table). Since the SPR data was based on binding to an individual CARD domain and the ApoptoAll model considers the heptameric apoptosome as a singular reactant, the SPR kinetic values were multiplied by seven to reflect the maximal availability of seven CARDs per apoptosome. In order to achieve a global parameter estimation approach, 100 different iterations of local parameter estimation were performed with local boundaries created using the lhsdesign function in Matlab for Latin Hypercube sampling.

Table 1: Upper and lower bounds used for the parameter estimation.

	Initial PC9 binding		Cooperative PC9 binding		C9 binding	
	k_{on} [μM^{-1} min^{-1}]	k_{off} [min^{-1}]	k_{on} [μM^{-1} min^{-1}]	k_{off} [min^{-1}]	k_{on} [μM^{-1} min^{-1}]	k_{off} [min^{-1}]
Upper bound	70	2	315	2	315	2
Lower bound	0	0	0	0	0	0

For the parameter estimation, simulated data was compared to two sets of experimental data with equal weighting using a least squares regression. Experimental data used was C3 substrate cleavage in HeLa cells, obtained previously from fitting a Boltzmann sigmoidal to FRET substrate data and the molecular timer data, extracted from Malladi and colleagues using ImageJ and normalised to the 5 minute time point(4,8). The cost function for each individual experiment was calculated via the least squares method with the following formula, where t is each time point with available experimental data:

$$cost = \sum_{t=1}^{n} \left(\text{model}(t) - \text{experiment}(t)\right)^2 \qquad [1]$$

The cost functions for both fits were then combined to a total cost:

$$cost_{\text{total}} = cost_{molecular\ timer} + cost_{substrate\ cleavage} \qquad [2]$$

The matlab function fmincon was used to minimise the cost function in each local estimation. The obtained optimal k-values were post-processed and the k-value combination chosen that fulfilled all of the following criteria:

k_{on} initial PC9 binding < k_{on} cooperative PC9 binding
k_{off} monomer PC9 unbinding > k_{off} dimer PC9 unbinding
k_{off} C9 unbinding > k_{off} dimer PC9 unbinding

The justification for this rule set is, in the case of cooperative recruitment, the k_{on} value of the initial PC9 molecule binding to the apoptosome should be considerably lower than the k_{on} value of the cooperative binding event. Furthermore, once PC9 is bound as a homodimer it is expected

to be more stable on the apoptosome and therefore the k_{off} value of cooperative binding should be lower than that of initial binding events. For the molecular timer to work it additionally is important that the k_{off} value of C9 is higher than the k_{off} value of PC9 and vice versa that the k_{on} value of C9 is lower than the k_{on} value of PC9. These assumptions are further supported by the SPR data extracted from Wu and colleagues, reported in Fig 2b. The resulting value sets were then chosen according to optimal cost function and implemented as ApoptoCoop.

Supplementary Figures:

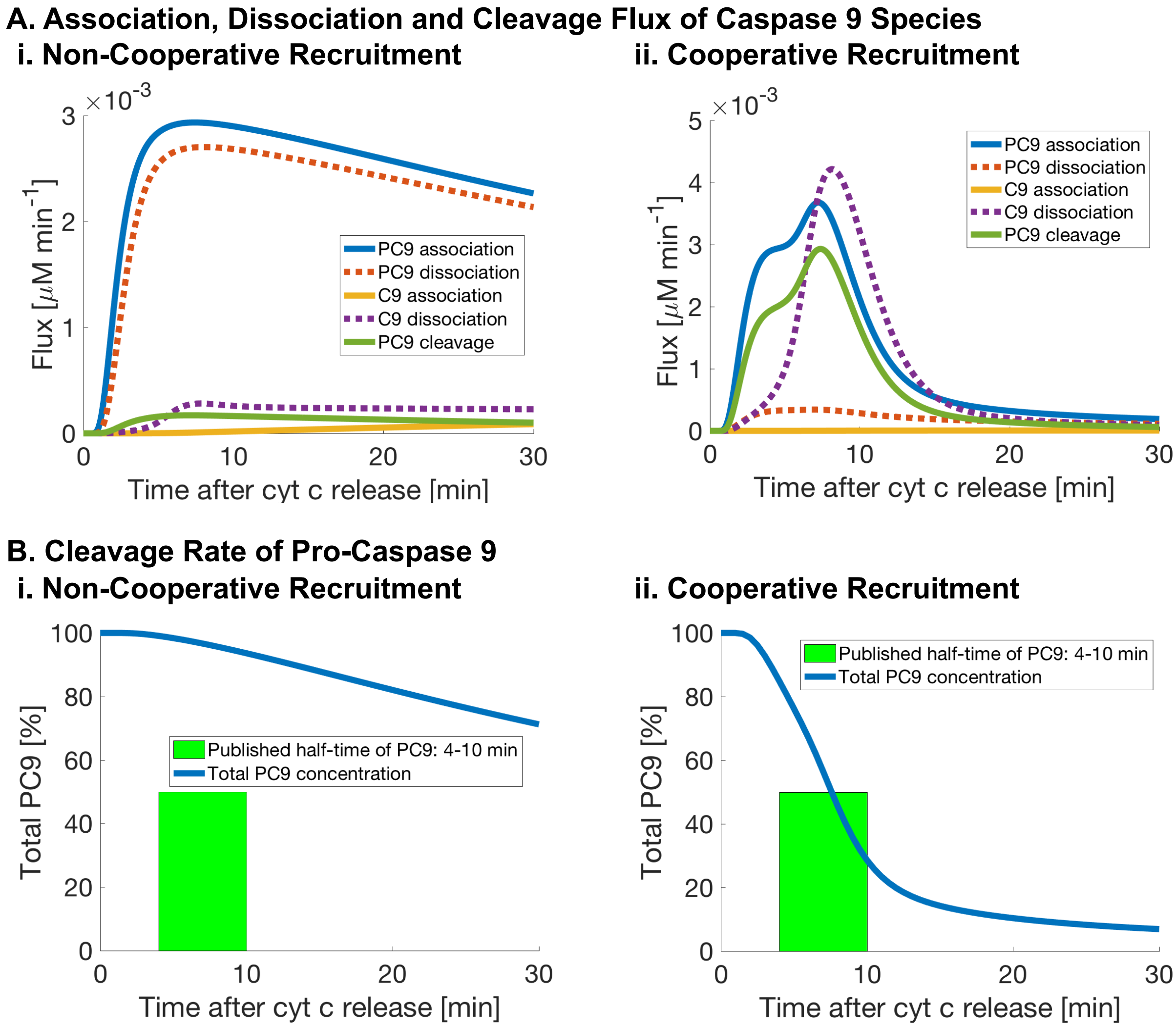

Supplementary Figure 1. A cooperative binding model of PC9 recruitment to the apoptosome reproduces experimental cleavage half-life of PC9 and leads to dissociation of C9. Flux analysis of association, cleavage and dissociation of PC9 and C9 at the apoptosome (A) using HeLa cell protein concentrations with non-cooperative recruitment(i) and cooperative

recuitment (ii). Simulated PC9 turnover rates against experimental rates from the literature (B) with non-cooperative recruitment(i) and cooperative recuitment (ii) (9–12).

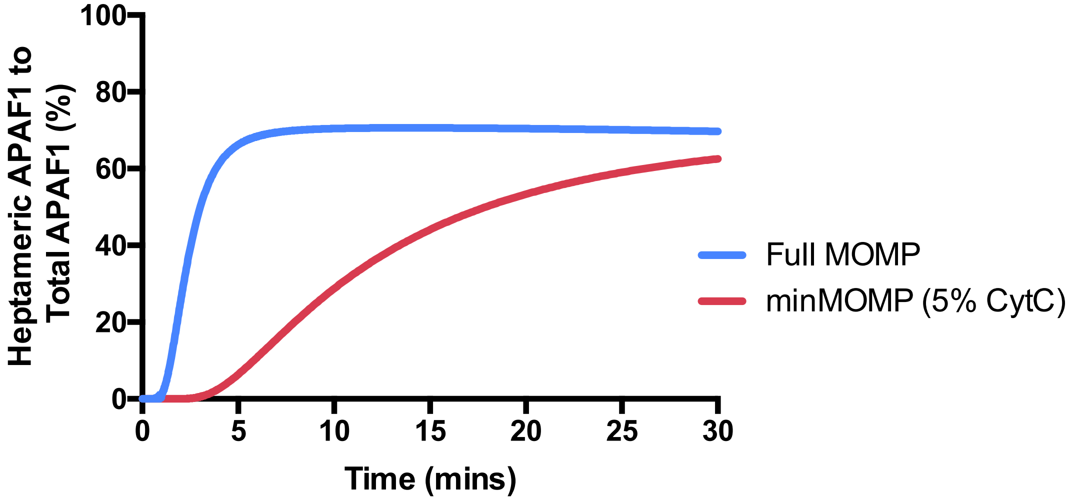

Supplementary Figure 2. Apoptosome formation efficiency after MOMP and minMOMP in HeLa cells. The formation of the heptameric apoptosome compared to total APAF1 after MOMP or minMOMP using conditions for HeLa cells.

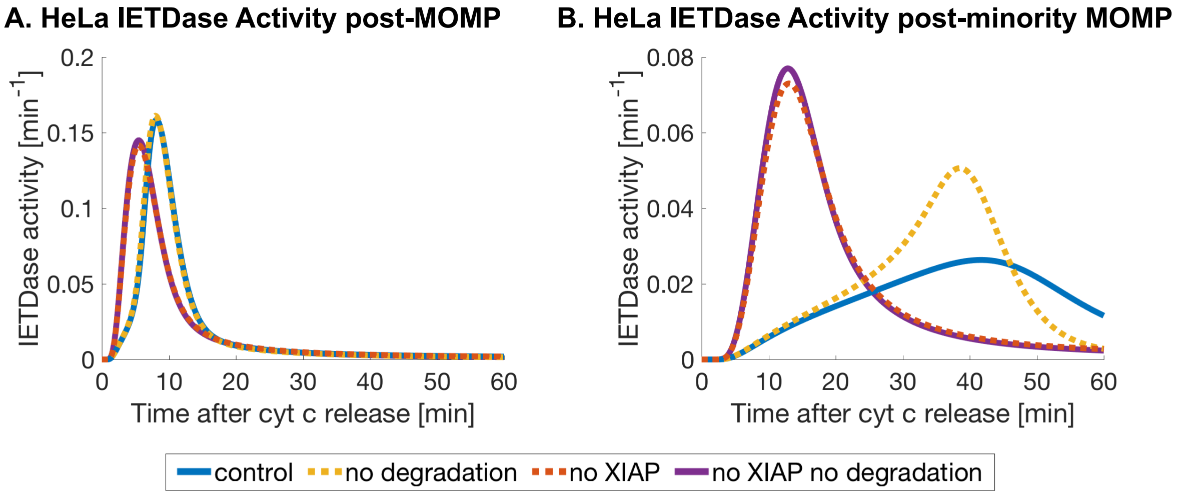

Supplementary Figure 3. Reduction of IETDase activity occurs in the absence of XIAP and degradation post-MOMP and minority MOMP. Traces of IETDase activity over time at the apoptosome in HeLa cells under normal control conditions, without XIAP and/or without protein degradation after MOMP (A) or minority MOMP (B).

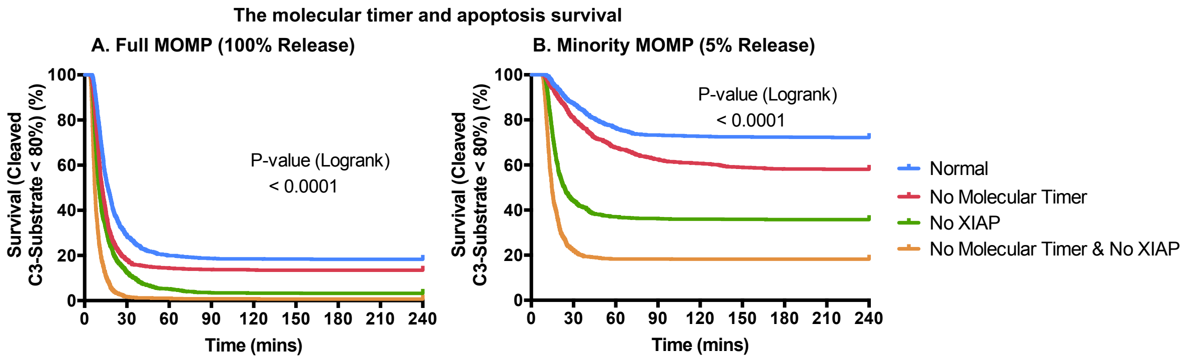

Supplementary Figure 4. The molecular timer can prevent apoptosis after MOMP and minority MOMP. Survival curves with and without the molecular timer for complete MOMP (A) and minority MOMP (B). Simulations were performed as in Fig4C, albeit with apoptotic cell death defined as C3-substrate cleavage of >80%. P-values in C from logrank test, n=1000 for all groups.

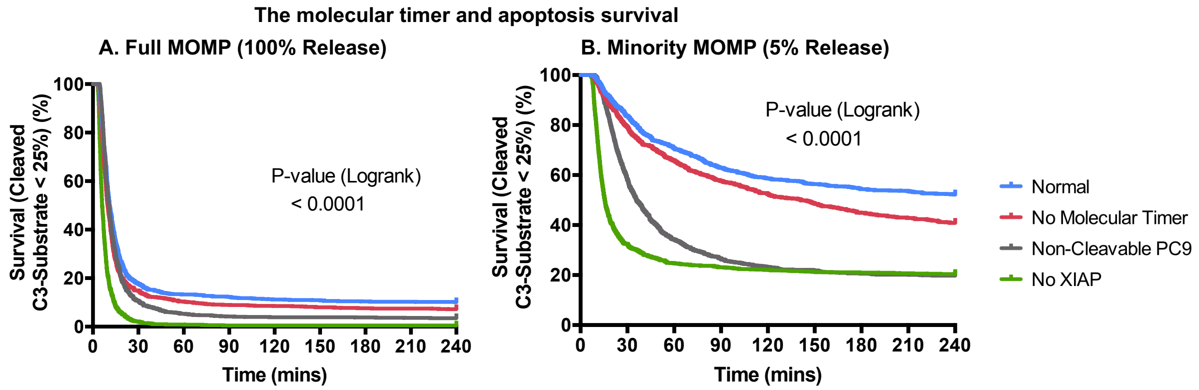

Supplementary Figure 5. Non-cleavable PC9 sensitises cells to apoptosis more than the molecular timer alone. Survival curves with and without the molecular timer, without XIAP and with non-cleavable PC9 for complete MOMP (A) and minority MOMP (B). P-values from logrank test, n=1000 for all groups.

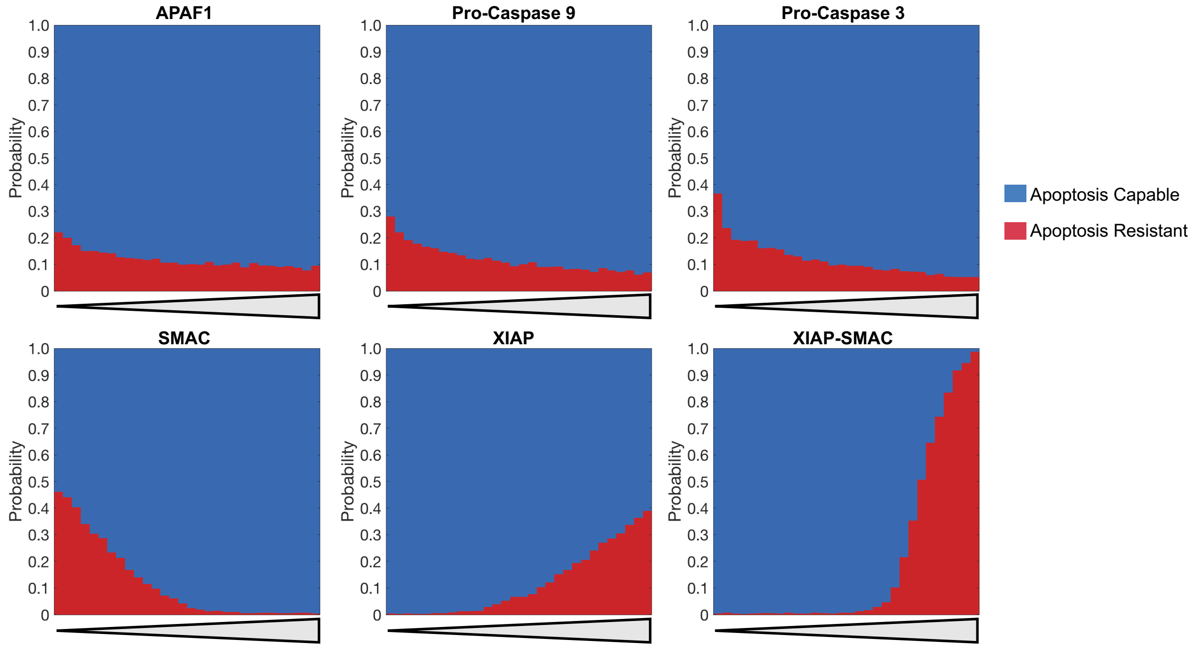

Supplementary Figure 6. The effect of protein concentrations on apoptosis decisions post-MOMP. The relative probability of being apoptosis capable or apoptosis resistant with increasing concentrations of apoptotic proteins after MOMP.

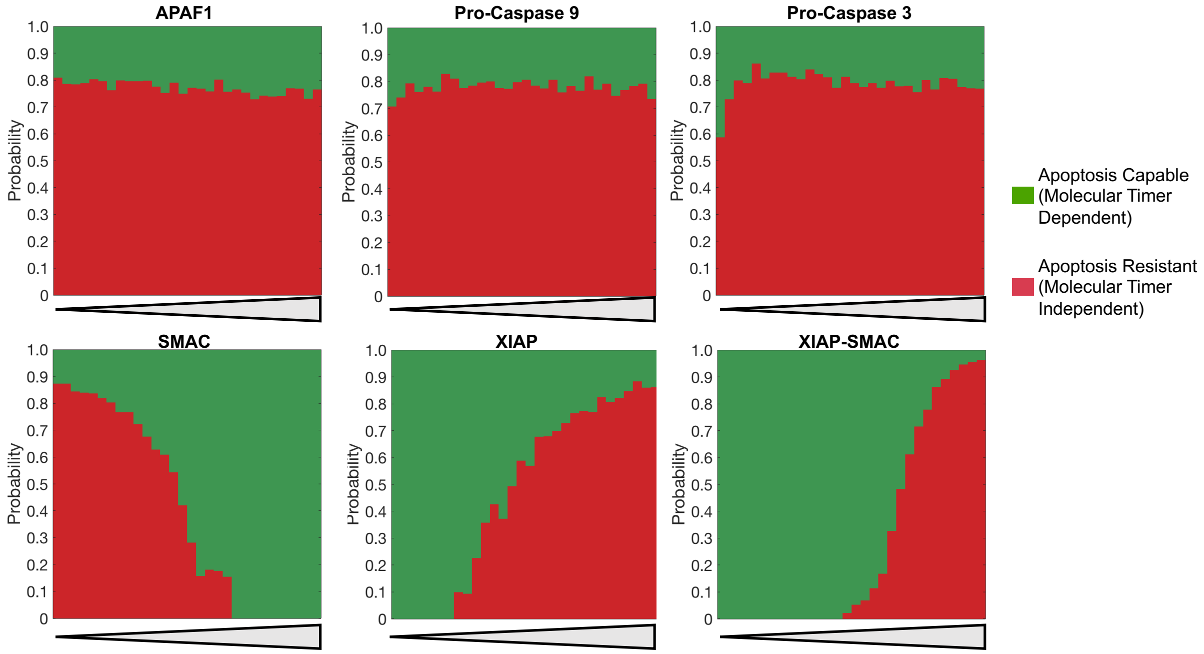

Supplementary Figure 7. The effect of protein concentrations on molecular timer-mediated apoptotic resistance post-MOMP. The relative probability of apoptosis resistance being molecular timer dependent or independent with increasing concentrations of apoptotic proteins after MOMP.

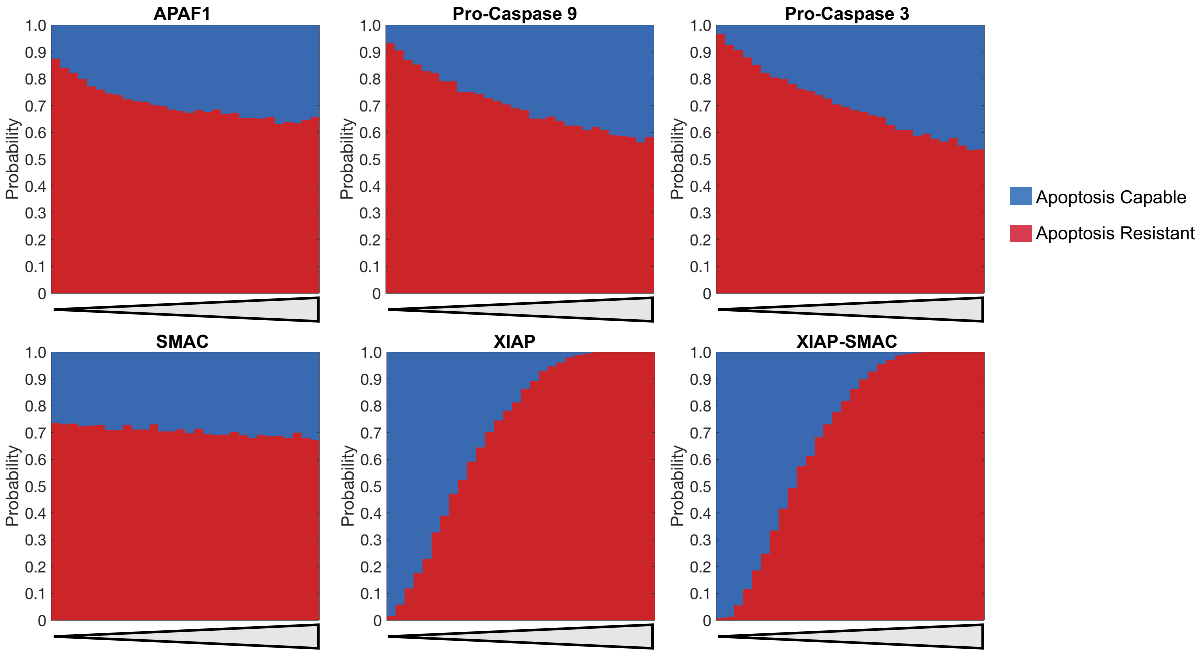

Supplementary Figure 8. The effect of protein concentrations on apoptosis decisions post-minority MOMP. The relative probability of being apoptosis capable or apoptosis resistant with increasing concentrations of apoptotic proteins after minority MOMP.

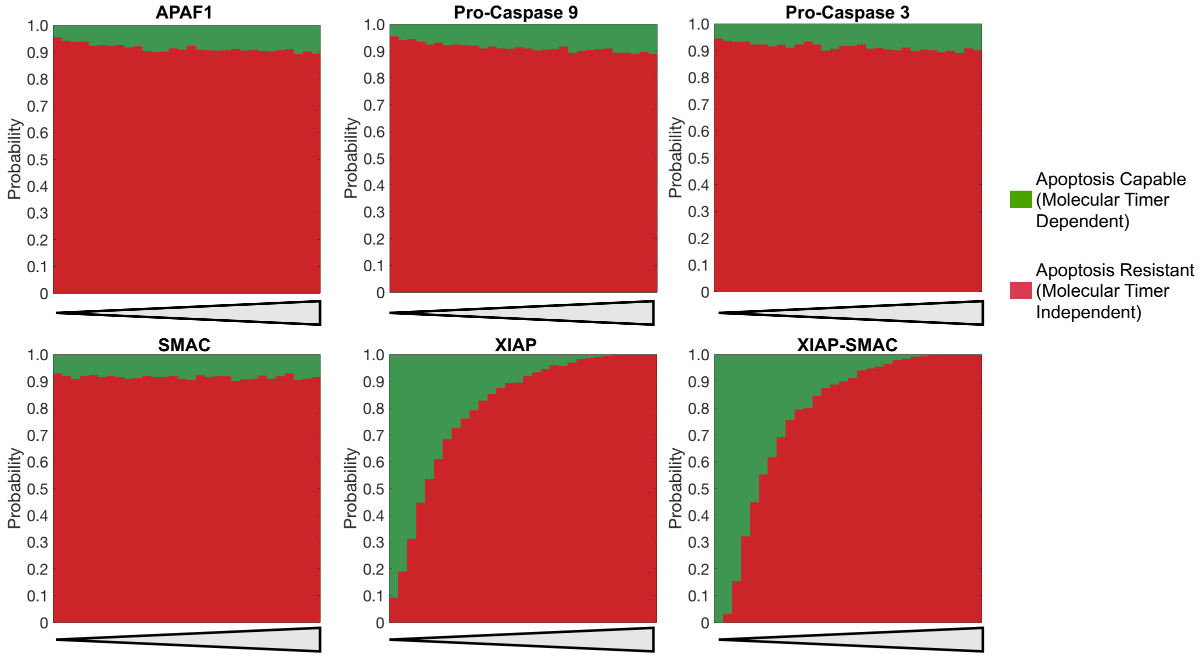

Supplementary Figure 9. The effect of protein concentrations on molecular timer-mediated apoptotic resistance post-minority MOMP. The relative probability of apoptosis resistance being molecular timer dependent or independent with increasing concentrations of apoptotic proteins minority MOMP.

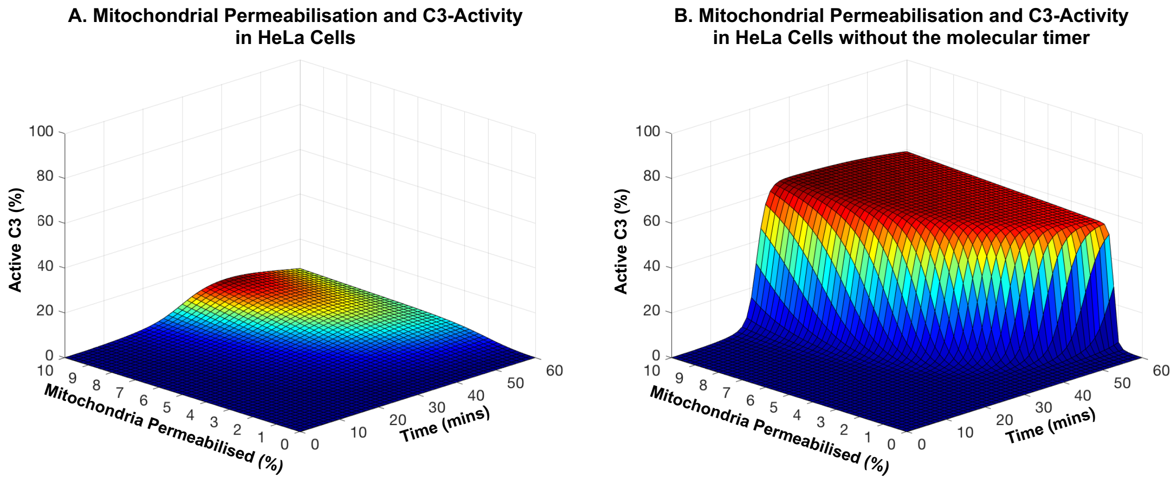

Supplementary Figure 10. The molecular timer may contribute to HeLa cell survival after minority MOMP. The level of free active C3 is plotted against time with different levels of mitochondria permeabilisation under normal conditions (A) or without the molecular timer (B).

References

1. Cain K, Bratton SB, Langlais C, Walker G, Brown DG, Sun X-M, et al. Apaf-1 Oligomerizes into Biologically Active ~700-kDa and Inactive ~1.4-MDa Apoptosome Complexes. J Biol Chem . 2000;275(9):6067–70.
2. Palacios-Rodríguez Y, García-Laínez G, Sancho M, Gortat A, Orzáez M, Pérez-Payá E. Polypeptide modulators of Caspase Recruitment Domain (CARD)-CARD-mediated protein-protein interactions. J Biol Chem. 2011;286(52):44457–66.
3. Würstle ML, Rehm M. A Systems Biology Analysis of Apoptosome Formation and Apoptosis Execution Supports Allosteric Procaspase-9 Activation. J Biol Chem . 2014;289(38):26277–89.
4. Rehm M, Huber HJ, Dussmann H, Prehn JHM. Systems analysis of effector caspase activation and its control by X-linked inhibitor of apoptosis protein. EMBO J. 2006;25(18):4338 LP – 4349.
5. Waterhouse NJ, Goldstein JC, Von Ahsen O, Schuler M, Newmeyer DD, Green DR. Cytochrome c maintains mitochondrial transmembrane potential and ATP generation after outer mitochondrial membrane permeabilization during the apoptotic process. J Cell Biol. 2001;153(2):319–28.
6. Mesner PW, Bible KC, Martins LM, Kottke TJ, Srinivasula SM, Svingen PA, et al. Characterization of Caspase Processing and Activation in HL-60 Cell Cytosol Under Cell-free Conditions: NUCLEOTIDE REQUIREMENT AND INHIBITOR PROFILE . J Biol Chem . 1999;274(32):22635–45.
7. Wu C-C, Lee S, Malladi S, Chen M-D, Mastrandrea NJ, Zhang Z, et al. The Apaf-1 apoptosome induces formation of caspase-9 homo- and heterodimers with distinct activities. Nat Commun. 2016;7:13565.
8. Malladi S, Challa-Malladi M, Fearnhead HO, Bratton SB. The Apaf-1•procaspase-9 apoptosome complex functions as a proteolytic-based molecular timer. EMBO J. 2009;28(13):1916–25.
9. Srinivasula SM, Ahmad M, Fernandes-Alnemri T, Alnemri ES. Autoactivation of

procaspase-9 by Apaf-1-mediated oligomerization. Mol Cell. 1998;1(7):949–57.
10. Zou H, Yang R, Hao J, Wang J, Sun C, Fesik SW, et al. Regulation of the Apaf-1/caspase-9 apoptosome by caspase-3 and XIAP. J Biol Chem. 2003;278(10):8091–8.
11. Hill MM, Adrain C, Duriez PJ, Creagh EM, Martin SJ. Analysis of the composition, assembly kinetics and activity of native Apaf-1 apoptosomes. EMBO J. 2004;23(10):2134–45.
12. Saikumar P, Mikhailova M, Pandeswara SL. Regulation of caspase-9 activity by differential binding to the apoptosome complex. Front Biosci. 2007;12:3343–54.
13. Rehm M, Dussmann H, Prehn JHM. Real-time single cell analysis of Smac/DIABLO release during apoptosis. J Cell Biol. 2003;162(6):1031–43.
14. Jiang X, Wang X. Cytochrome c promotes caspase-9 activation by inducing nucleotide binding to Apaf-1. J Biol Chem. 2000;275(40):31199–203.
15. Reubold TF, Wohlgemuth S, Eschenburg S. A new model for the transition of APAF-1 from inactive monomer to caspase-activating apoptosome. J Biol Chem. 2009;284(47):32717–24.
16. Purring-Koch C, McLendon G. Cytochrome c binding to Apaf-1: the effects of dATP and ionic strength. Proc Natl Acad Sci. 2000;97(22):11928–31.
17. Purring C, Zou H, Wang X, McLendon G. Stoichiometry, free energy, and kinetic aspects of cytochrome c: Apaf-1 binding in apoptosis. Vol. 121, Journal of the American Chemical Society. American Chemical Society; 1999. p. 7435–6.
18. Cain K, Bratton SB, Langlais C, Walker G, Brown DG, Sun XM, et al. Apaf-1 oligomerizes into biologically active ~700-kDa and inactive ~1.4-MDa apoptosome complexes. J Biol Chem. 2000;275(9):6067–70.
19. Pop C, Timmer J, Sperandio S, Salvesen GS. The Apoptosome Activates Caspase-9 by Dimerization. Mol Cell. 2006;22(2):269–75.
20. Stennicke HR, Renatus M, Meldal M, Salvesen GS. Internally quenched fluorescent peptide substrates disclose the subsite preferences of human caspases 1, 3, 6, 7 and 8. Biochem J. 2000;350 Pt 2:563–8.
21. Timmer JC, Zhu W, Pop C, Regan T, Snipas SJ, Eroshkin AM, et al. Structural and kinetic determinants of protease substrates. Nat Struct Mol Biol. 2009;16(10):1101–8.
22. Riedl SJ, Renatus M, Schwarzenbacher R, Zhou Q, Sun C, Fesik SW, et al. Structural Basis for the Inhibition of Caspase-3 by XIAP. Cell. 2001;104(5):791–800.
23. Huang Y, Rich RL, Myszka DG, Wu H. Requirement of both the second and third BIR domains for the relief of X-linked inhibitor of apoptosis protein (XIAP)-mediated caspase inhibition by Smac. J Biol Chem. 2003;278(49):49517–22.
24. Ferraro E, Pulicati A, Cencioni MT, Cozzolino M, Navoni F, di Martino S, et al. Apoptosome-deficient cells lose cytochrome c through proteasomal degradation but survive by autophagy-dependent glycolysis. Newmeyer DD, editor. Mol Biol Cell. 2008;19(8):3576–88.
25. Eissing T, Conzelmann H, Gilles ED, Allgöwer F, Bullinger E, Scheurich P. Bistability analyses of a caspase activation model for receptor-induced apoptosis. J Biol Chem. 2004;279(35):36892–7.
26. Yoo SJ, Huh JR, Muro I, Yu H, Wang L, Wang SL, et al. Hid, Rpr and Grim negatively regulate DIAP1 levels through distinct mechanisms. Nat Cell Biol. 2002;4:416.
